# Arena-Anchored Urban Development Projects and the Visitor Economy

**DOI:** 10.3389/fspor.2022.912926

**Published:** 2022-06-21

**Authors:** Taryn Barry, Daniel S. Mason, Robert Trzonkowski

**Affiliations:** Faculty of Kinesiology, Sport, and Recreation, University of Alberta, Edmonton, AB, Canada

**Keywords:** cities, arenas, stadiums, visitor economy, placemaking

## Abstract

Cities of all sizes are actively engaged in developing various urban infrastructure projects. A common strategy used in larger North American cities is employing arena-anchored urban development projects, where a professional sports team is used as an anchor tenant of a sports facility to generate development in the city. One means of relocating economic activity is to increase visitation to the desired redevelopment area. In this paper we used the visitor economy as a lens to explore how arena-anchored projects and the professional sports teams that play there fit into a local city's tourism economy. To conduct this study, a multi case study design was used to draw data from two cities: Columbus, Ohio, and Detroit, Michigan. Interviews were goal directed and conducted in person with leaders in Columbus (*n* = 9) and Detroit (*n* = 10), and inductive and deductive approaches to coding were undertaken in the form of content analysis. The results indicate that growing the visitor economy through arena anchored urban development relies on planned placemaking via the strategic approach of bundling diverse amenities together. These findings provide valuable feedback to those cities considering arena development projects, and how the arenas may be combined with other civic amenities to undergird the local visitor economy.

## Introduction

Cities and regions of all sizes globally are actively engaged in developing and building various urban infrastructure including amenities such as museums, convention centers, and sports and entertainment facilities (Rosentraub, 2010). A common strategy used in larger North American cities, is employing arena-anchored development projects, where a professional sports team is used as an anchor tenant of a facility to generate greater development in the city. These development projects remain prominent in urban redevelopment planning for many cities, and have had both their supporters and detractors. There are several rationales that city leaders and business leaders provide for the use of public funds to build new sports facilities and attract professional sport teams, such as economic and community development, improving quality of life of residents, and tourism (Baade and Matheson, 2004; Chalip, 2006; Misener and Schulenkorf, 2016). However, it is largely viewed as a contested practice as independent academic research largely debunked the purported economic benefits of hosting teams decades ago (Quirk, 1987; Crompton, 1995; Baade, 1996; Coates and Humphreys, 2008). Despite such concerns, cities continue to allocate public funds and build facilities, often as part of comprehensive downtown (re)development efforts, in an attempt to relocate economic activity back to the city core (Mason, 2016).

One rationale for investing is to increase visitation to the redevelopment area; in this paper, the visitor economy is used as a lens to specifically explore how arena-anchored urban development projects and the professional sports teams that play there fit into a local city's tourism economy. At the core of the visitor economy is the assumption that economic activity comes from various types of visitors (Reddy, 2006). The visitor economy is a broad term that recognizes the economic activity of all visitors to a destination, such as business travelers, those visiting friends and family members, students, and people attending sporting and cultural events (Hristov, 2015). It also considers all the elements that make a destination successful in terms of visitation. This includes the natural environment; heritage, culture, and iconic buildings; retail, sport, leisure, and cultural facilities; restaurants and hotels; transportation and parking; the services that make the place clean, safe, and welcoming; and the infrastructure that make it an accessible place to visit while shaping its sense of place (Reddy, 2006).

Sense of place, and its anticipated outcome of placemaking (Aravot, 2002) is conceptualized by several academic disciplines. Placemaking in urban studies has shifted from focusing on the physical elements of projects (Day, 1992) to a democratic intervention between all stakeholders (Shibley et al., 2003). Placemaking is viewed as a process and a means to an end; the end being the creation of quality places (Wyckoff, 2014). It is how a cultural group marks its values, perceptions, memories, and traditions on a geographic space and offers meaning to the landscape (Coates and Seamon, 1984; Othman et al., 2013). It should also be understood from both organic and planned perspectives and as an essential part of tourism destination development (Lew, 2017). While organic placemaking evolves from local, bottom-up initiatives, planned placemaking is a top-down approach that contains modern, predictable, and contrived features, designed for mass tourism consumption (Lew, 2017). It is also the most common approach used in arena anchored urban development projects. Lew (2017) reasoned that for planned placemaking to create a convincing sense of place, there must also be some space for the evolution of organic placemaking to occur.

Moreover, Richards and Duif (2019) proposed that planned placemaking only works effectively if three elements are combined with equal stakeholder support. These elements include: (1) the tangible and intangible resources available (i.e., capital, land, human, or infrastructure), (2) the meanings linking people and stakeholders with the places they live in (i.e., symbols, identity markers, values, memories, or traditions), and (3) the creative and innovative use of resources and meanings that capture the public's attention (i.e., narratives, storytelling, branding). The third element of planned placemaking may be an important device for cities to increase their competitive advantage amongst other cities (Barney, 1991).

City leaders may perceive the strategic approach of bundling a diverse amenity mix as a means to successful planned placemaking. Natural and constructed amenities that are planned in proximity to one another may contribute to placemaking as well as bridge other amenities or nearby neighborhoods as part of this amenity mix. Natural physical amenities can consist of water, climate, humidity, environmental attractiveness, while constructed amenities can consist of museums, convention centers, coffee shops, juice bars, and research libraries (Clark, 2004). City and business leaders may perceive and advocate for the bundling of amenities near one another as the key to successful arena anchored development and broader urban re(development). There is limited research to support strategically bundling amenities for successful outcomes, therefore we draw from the existing research on the bundling of sport events, tourism, heritage, and hospitality products. For instance, Chalip and McGuirty (2004) reasoned an effective way to incorporate sport events more strategically into the host destination's broader tourism product and service mix, is to bundle sport event components with the host's current attractions via a “mixed bundling strategy” (p. 267). Meanwhile, Xu et al. (2016) investigated event bundling strategies from the perspective of various event stakeholders, illustrating that attendees' perceived experiences were enriched by attending multiple events over the course of one trip. In addition, Huang et al. (2016) contended that rural communities have shown to improve secondary attractions and diversify their tourism product by bundling heritage attractions with non-heritage activities (Huang et al., 2016). This research has informed this study as we sought to understand how planned placemaking via a mixed bundling strategy can be essential to developing the visitor economy. More specifically, this study investigated how stakeholders in two North American cities – Detroit, Michigan and Columbus, Ohio – utilize conceptualizations of placemaking to develop their broad visitor economy via arena-anchored urban development initiatives. These two cases represent examples of existing comprehensive development projects that have had varying degrees of success, in cities not extensively viewed as tourism destinations.

## Method

To conduct this study, a multi case study design (Eisenhardt, 1989) was used to draw data from two cities: Columbus, Ohio and Detroit, Michigan. For more context, the city of Columbus is the 14^th^ largest city in the United States and continues to be the fastest growing Midwest city (US Census Bureau, 2020a) in contrast to other major cities in Ohio such as Cleveland and Cincinnati. The *Columbus Arena District* is widely regarded as a success story for sports- facility anchored urban development. For example, Columbus' amenities strategy was found in the discourse surrounding the construction of a new arena-anchored district in Edmonton, Canada (Sant et al., 2019), acknowledging Columbus as a successful exemplar of integrated urban development comprising of commercial, residential, hospitality, and entertainment development in a mid-sized city (Rosentraub, 2014). However, despite Columbus' perceived success, it is difficult to attribute increased land values, intangible benefits and increased economic activity solely to a specific facility or sports team; as a result, there is still skepticism associated with sports facility-anchored development projects within the academic community (Propheter, 2019).

Meanwhile, Detroit provides an interesting site to explore sports facilities and urban development since it remains one of the most blighted cities in North America despite recent attempts to revitalize its downtown core. While the city of Detroit is the 27^th^ largest city in the United States and the largest city in the state of Michigan, it has had a consistently declining population for the past decade (US Census Bureau, 2020b). Detroit was known for its major role in the global automobile industry but has moved to other strategic options to sustain the local economy (Galster, 2012). The recent arena-anchored development *District Detroit* is a sports and entertainment development anchored by Little Caesars Arena, a multi-sport facility that opened in 2017. While the *Columbus Arena District* is praised, *District Detroit* has been criticized for gentrification that disproportionately affects Detroit's African American population, as well as delayed plans on proposed residential and hotel development, and historic buildings restoration (Pinho and Shea, 2019; Graham et al., 2021).

Nine interviews with nine individuals were conducted in Detroit in December 2018, while nine interviews with thirteen individuals were conducted in Columbus in February 2019. Prior to conducting semi structured interviews (Merriam, 1988) participants were recruited by researching prominent leaders in Detroit and Columbus online and sending introductory letters via email. In other words, interviewees were sought who would likely espouse and express the kinds of narratives associated with their respective cities and arena projects of interest to this study. A snowball sampling technique (Goodman, 1961) was used after initial interviews, as respondents were asked to identity other actors with whom they were linked to Rowley (1997).

Table 1 presents the characteristics – such as job title and the sector of work – for each subject that participated in this study, which included local civic leaders, business stakeholders, journalists, city and county administrators, facility operators, an urban planner, academic consultants, and executives with the local chamber of commerce and sports commissions.

**Table 1:** Participant characteristics.

**Job title and organization**	**Field of work**
**City of Columbus**	
Executive Director, Franklin County Convention Facilities Authority	City administration 5
President and CEO, Columbus Chamber of Commerce	City administration 1
Director, Franklin County Economic and Development Department	City administration 2
Business Editor, The Columbus Dispatch	Journalism/print media 1
City Hall Reporter, The Columbus Dispatch	Journalism/print media 2
Executive Director, Greater Columbus Sport Commission	Non-profit 1
Director of Public Relations, Experience Columbus	Tourism/hospitality
Director of Events, Greater Columbus Sports Commission	Non-profit 2
Director of Marketing, Greater Columbus Sports Commission	Non-profit 3
City Auditor, City of Columbus	City administration 3
Director, Department of Development, City of Columbus	City administration 4
Vice President, Planning, Architecture and Real Estate, The Ohio State University	Urban planning
Associate Vice President of Business Advancement, The Ohio State University	Facility management and operation
**City of Detroit**	
Chief of Staff, Detroit Economic Growth Corporation	City administration 1
Chief Financial Officer and Executive Vice President of Administration, Detroit Economic Growth Corporation	Non-profit 1
Director of the Detroit Sports Commission	Non-profit 2
Director for the Department of Civil Rights, Inclusion, and Opportunity, City of Detroit	City administration 2
Vice President of Sales, Marketing, and Sports for the Detroit Metro Convention and Visitors Bureau	Tourism/hospitality
Projects Reporter and past Business Writer for the Detroit News	Journalism/print media
Elected Council representative of District 6, City Council	City administration 3
Professor at University of Michigan	Academia 1
Dean at the Mike Ilitch School of Business at Wayne State University	Academia 2

In person interviews lasted approximately 1 h and were guided by semi structured interview questions. The Detroit interviews produced 215 single spaced pages of transcribed interview text and the Columbus interviews produced 230 single spaced pages. Once the interviews were transcribed, participants were emailed transcripts and provided an opportunity to request omission of specific text.

Data were coded using a form of content analysis, which is comprised of a systematic, theory-driven approach to texts, examining both latent and manifest content (Mayring, 2000). Through an adapted method of analysis derived from both Mayring (2000) and Denis et al. (2001), both inductive and deductive approaches to coding were undertaken.

## Results

The results of this study indicate that city stakeholders assert that advancing and growing the visitor economy through arena-anchored urban development relies on planned placemaking via one explicit strategy, which is the approach to bundling a variety of unique amenities in proximity together. This reveals that sport and professional sports teams are not central to placemaking but are the anchor amenity that attracts other desirable amenities and their visitors. Results from the data analysis will be presented in more detail below, followed by a discussion and the implications of the results.

### Detroit, Michigan

In Detroit, a Non-Profit (1) official shared the significance of how diverse amenities entice visitors to the downtown core, stating:

So again.....attracting more conventions, more visitors, more bodies down to the downtown which is good for the restaurants, it's great for the hotels and we've got a hotel boom going on. You know the old fire house was converted into a new hotel.

While a city official (3) discussed the need to contain development in close proximity to one another as a means to attract people to visit, work and live in the District, arguing:

Beyond that what my hope is, is that it is going to create a framework where, I don't know how familiar you are with the arena neighborhood itself but it's a 40-square block neighborhood that's contained within the quote, unquote District. But the neighborhoods surrounding that need additional development, too. So I think the hope is that with the progressive development of The District that the neighborhoods surrounding The District also will start to see more investment because there is going to be this additional kind of corridor now where more people will be living and working.

In addition, a senior tourism/hospitality manager discussed how the District was a leisure destination that has attracted and integrated various restaurant amenities:

Whoever would've thought that we'd be a you know, a leisure destination, but with all of the buzz and the conversation changing about Detroit, we've had so many people that have really come to experience it and find out what's going on, you know we've had over 100 restaurants that have opened over the last 3 years.

Finally, a city official (1) refereed to “commercial corridors” whereby neighborhoods are revitalized via a mixed amenity bundling strategy, explaining:

We're definitely seeing some good things happen in the neighborhoods and we're seeing developers that are willing to take more of a risk in neighborhoods than they were certainly 10 years ago and even 5 years ago, but we have a lot of work to do within Detroit neighborhoods both from an affordable housing standpoint and also in the revitalization of our smaller commercial corridors.

### Columbus, Ohio

Meanwhile in Columbus similar sentiments were expressed. One city official (5) articulated how bridging amenities and neighborhoods together is mutually beneficial for all stakeholders:

We're sitting in the Convention Center now and the Convention Center is sandwiched between the arena district and the Short North Arts District and so it makes this center competitive, that adjacency and proximity, that walkability is what we call it makes it attractive to conferences and trade shows and conventions that rotate around the region and rotate around North America. And so it is the entertainment, the restaurants, the bars, the shopping opportunities in both the Arts District and the Arena District that contribute to the success of the convention, tourism, and trade, and visitor economy here. It's symbiotic.

This same city official (5) conveyed the significance of revitalizing a contaminated brownfield with a sports arena as the anchor and catalyst for economic growth, stating:

....here the arena which is now 20 years old did in fact spark growth and development of an arena district surrounding it, a mixed-use district of residential and commercial and entertainment, lots of jobs, lots of property value created, a lot of economic activity created and it has anchored that. The master developer refers to it as a mixed-use district masquerading as a sports and entertainment district. It has succeeded in anchoring a site that was formerly a brown site, brown field, formerly a penitentiary, which was with lots of contamination and lots of ugly history associated with it. It blocked the central business district from growth and development and so its removal and replacement with the arena and the arena district that surrounded it has succeeded in keeping the urban core healthy and growing.

Furthermore, a non-profit administrator (1) shared the potential benefits of bundling amenities in close proximity to one another as safety, cleanliness, walkability, and restaurant variety, stating:

I'm really proud of the density of the walkability so you're not just walking up one street where there's 30 restaurants but, you know, there's really a hundred restaurants of varying price points and I think people still feel very safe. I'm not saying we wouldn't have a mishap here and there but by and large we're known for the safety piece of it, the cleanliness, and the fact that there's such a variety, so there really is, you wanna go to a sports bar? Great, do you wanna go to a French restaurant? Sure. So I think what I love about it best is that people have so many options and they come here and they really can sort of personalize their stay.

A senior level urban planner elaborated on the way Nationwide arena anchored other inimitable amenities that not only bridged the downtown core to an neighborhood called the Short North, but also strengthened the arts community, restaurant scene, and Columbus' city national brand, arguing:

…so the Short North, if you go research it, the *New York Times* did an article, it's been a few years back now, where they said the Short North is the single best homegrown arts community in the country and at the time we had more galleries in there but as rents have gone up the only thing that can pay the rents are the restaurants and so it's become like a restaurant mecca now but yeah so we started to get some real positive publicity, like unsolicited. So the *New York Times* articles and it just kept building and I think it was largely the Arena District put it on the map, got a lot of ink for that.... the Arena District was really the big bang that was the origin of that.

### Strategic Approach to Bundling Amenities

Respondents in Columbus commonly referred to their bundling strategy in relation to the public-private partnership called *The Columbus Way*. For example, one city administrator (4) contended:

You'll hear some talk about, I don't know if in the research you've come across this phrase The Columbus Way where we're doing public-private partnerships and it really does come down to basically shared values. You know shared belief that the government and the private sector aren't adversarial. That what's good for one is good for the other, so long as you're focused on inclusion, quality of life, and strong governments.

While a senior level urban planner in Columbus reasoned every city has unique features that must be considered when developing and implementing a strategy for urban planning, stating:

....every city's got its own levers that you have to pull but you have to figure'em out what the levers are and then what becomes critical is you have to know the order to pull'em and that's trickier. So it takes a strategy and I can't say that we set out with a strategy but I would say that Columbus, as the strategy became apparent they didn't deviate from it… Which is one reason they're teaching a course in Harvard on it.

This defined strategy to building amenities did not emerge in the Detroit data as it did in Columbus, yet it was found that respondents in Detroit considered the importance of future narratives in relation to District Detroit and their broader downtown redevelopment plans.

## Discussion

The results of this study reveal city stakeholders feel that planned placemaking via bundling amenities may be essential to developing the visitor economy in urban centers when it concerns arena and stadium projects. As such, this paper illuminates new understandings of how North American cities and their stakeholders may employ planned placemaking to develop their broad visitor economy via arena-anchored urban development initiatives.

First, professional sports teams are not viewed as the central feature of placemaking to city stakeholders but rather viewed as the anchor that can initiate placemaking through attracting other amenities to the area, and their visitors. Analysis suggests that other, intangible benefits, are secondary to the goal of tourism and economic development. This is illustrated through the description of multi-visitation strategies through a diverse amenity mix, including corporate and leisure travel, sport and international tourism, youth sport travel, experiential tourism, niche tourism (i.e., stadium specific travel), as well as resident/local visitation.

The results also show the extent to which the development of arena districts is a strategy cities and local stakeholders utilize to increase visitation in various forms under an umbrella of creating economic impact. In Columbus, respondents suggested their strategy was informed by their public-private partnership called *The Columbus Way*, which is described as shared values of community stewardship and progress (Columbus Partnership, 2021). This finding illustrates that for the respondents in Columbus, this partnership was crucial for the placemaking success of the Columbus Arena District. While their amenities bundling approach may have evolved organically to where it is now it is a deliberate strategic approach, it may be unique to Columbus making it a complicated process for other cities to adopt.

That being said, other cities referring to the success of Columbus to support their own arena anchored development plans can observe that Columbus does not possess any tangible and unique competitive advantages that contribute to placemaking, in comparison to the placemaking success that a city such as Barcelona has had since hosting the 1992 Olympic Games (Mansilla and Milano, 2019). One reason why Columbus may have had success in developing this amenities bundling approach is that it was not battling against a negative brand image. Meanwhile, post-industrial cities like Detroit are attempting to reinvent their city brand as it was once known for its major role in the global automobile industry. This may mean that placemaking strategies may be more difficult for these latter cities.

Finally, respondents in each city highlighted the importance of meeting high quality-of-life indicators for both the residents *and* the visitors to the region. This challenges existing literature that contends tourism development is independent of the interest of residents (Eisinger, 2000). This finding contributes to the existing research on the visitor economy by presenting the new understanding that city stakeholders today are planning for - and seeking out - projects that will improve quality of life of both the visitors and residents.

By examining cases of various degrees of success and stages of completion, this paper provides valuable feedback to those cities considering arena development projects in their respective cities, and how the arenas may be combined with other civic amenities to undergird the local visitor economy. The success of arena districts appears to rest on the multi-faceted approach of planned placemaking via bundling a variety of leisure, sport, and entertainment amenities in a concentrated area. Therefore, cities looking to use arenas or stadiums to anchor further urban development should consider their resource and stakeholder capacities and needs to complete such a project successfully.

## Data Availability Statement

The original contributions presented in the study are included in the article/supplementary material, further inquiries can be directed to the corresponding author/s.

## Ethics Statement

The studies involving human participants were reviewed and approved by the University of Alberta Research Ethics Office. The patients/participants provided their written informed consent to participate in this study.

## Author Contributions

DM, TB, and RT collected the data. TB analyzed the data. TB and DM wrote the article. All authors contributed to the article and approved the submitted version.

## Funding

This work was supported by Social Sciences and Humanities Research Council (SSHRC) Insight Grant: #435-2015-0985.

## Conflict of Interest

The authors declare that the research was conducted in the absence of any commercial or financial relationships that could be construed as a potential conflict of interest.

## Publisher's Note

All claims expressed in this article are solely those of the authors and do not necessarily represent those of their affiliated organizations, or those of the publisher, the editors and the reviewers. Any product that may be evaluated in this article, or claim that may be made by its manufacturer, is not guaranteed or endorsed by the publisher.
